# An exotic parasitoid provides an invasional lifeline for native parasitoids

**DOI:** 10.1002/ece3.2577

**Published:** 2016-12-20

**Authors:** Joanna K. Konopka, Tim Haye, Tara Gariepy, Peter Mason, David Gillespie, Jeremy N. McNeil

**Affiliations:** ^1^ Department of BiologyWestern UniversityLondonONCanada; ^2^ London Research and Development CentreAgriculture and Agri‐Food CanadaLondonONCanada; ^3^ CABI‐Europe SwitzerlandDelémontSwitzerland; ^4^ Ottawa Research and Development CentreAgriculture and Agri‐Food CanadaOttawaONCanada; ^5^ Agassiz Research and Development CentreAgriculture and Agri‐Food CanadaAgassizBCCanada

**Keywords:** biological control, brown marmorated stinkbug, egg parasitoids, evolutionary trap, facultative hyperparasitoid, larval competition

## Abstract

The introduction of an exotic species may alter food webs within the ecosystem and significantly affect the biodiversity of indigenous species at different trophic levels. It has been postulated that recent introduction of the brown marmorated stinkbug (*Halyomorpha halys* (Stål)) represents an evolutionary trap for native parasitoids, as they accept *H. halys* egg masses as a host but produce no viable progeny. Interspecific interactions between European egg parasitoid, *Trissolcus cultratus* (Mayr), and an Asian parasitoid, *Trissolcus japonicus* (Ashmead), were assessed by providing egg masses to *T. cultratus* at various time intervals following the initial parasitization by *T. japonicus*. The suitability of the host for the parasitoid development was re‐assessed by providing *T. cultratus* with fresh and frozen egg masses of various ages. The likelihood of *T. cultratus* being able to attack previously parasitized egg masses was determined by assessing the duration of egg mass guarding behavior by *T. japonicus* following parasitization. The results of experiments examining the interspecific interactions between a native European egg parasitoid, *T. cultratus*, and an Asian parasitoid, *T. japonicus* (a candidate for the biological control of *H. halys*), showed that the native species can act as facultative hyperparasitoid of the exotic one. Although this is only possible during certain stages of *T. japonicus* development, the presence of the introduced parasitoid may reduce the impact of the evolutionary trap for indigenous parasitoid species. There is a possibility that the occurrence of facultative hyperparasitism between scelionid parasitoids associated with stinkbugs is common. This resulting intraguild predation could promote conservation and stabilization of natural communities by impacting the diversity and population dynamics of native stinkbugs and their parasitoids (e.g., by allowing native parasitoids to avoid wasting reproductive effort on unsuitable hosts), or reduce success of biological control programs (e.g., by reducing the population size of the exotic parasitoids).

## 1 Introduction

Invasive species may pose major threats to the biodiversity of native species at the same or higher trophic levels due to competition, predation, or facilitation (Elton, 2000; Pimentel et al., 2000; Rodriguez, 2006; Schmitz & Simberloff, 1997; Simberloff, 1981). Furthermore, the establishment of one exotic species can facilitate the establishment and spread of additional nonindigenous species, potentially leading to synergistic, negative effects, referred to as “invasional meltdown” (Simberloff, 2006; Simberloff & Von Holle, 1999). In contrast, the establishment of an invasive species may benefit a native one in a number of different ways, such as being a “trophic subsidy” by providing a new exploitable resource for native species at a higher trophic level (Rodriguez, 2006).

Indigenous natural enemies will benefit from the presence of an exotic host if their survival and/or reproduction is enhanced by exploiting exotic species as a trophic subsidy. Conversely, if previously reliable cues are no longer associated with adaptive outcomes following the invasion of nonindigenous species, this could result in an evolutionary trap that reduces the fitness and reproductive success of the native organism (Berthon, 2015; Schlaepfer, Runge, & Sherman, 2002; Schlaepfer et al., 2005). If a native parasitoid accepts an invasive species as a host, but fails to complete development, then the host becomes an evolutionary trap for the native species (Abram et al., 2014; Schlaepfer et al., 2002). However, an evolutionary trap is not necessarily inescapable (Berthon, 2015) and may be circumvented if the organism learns avoidance behavior, or develops mechanical and/or physiological means that permit it to exploit the host (Keeler & Chew, 2008; Phillips & Shine, 2004).

**Figure 1:**
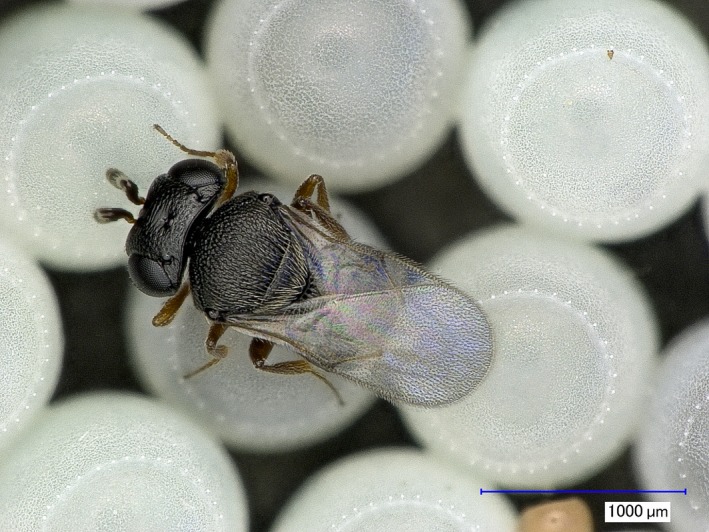
Female of *Trissolcus cultratus* on *Halyomopha halys* egg mass

The potential of an evolutionary trap exists with the widespread establishment of the invasive pest, *Halyomorpha halys* (Stål) (Hemiptera: Pentatomidae), in Europe and North America because *H. halys* eggs are readily attacked by native *Trissolcus* Ashmead and *Telenomus* Haliday parasitoid wasp species (Hymenoptera: Scelionidae) (Figure 1), but they are unsuitable for development of larvae of these parasitoids (Abram et al., 2014; Haye et al., 2015). The impact of this evolutionary trap for native parasitoids may be further exacerbated by the proposed introduction of the Asian parasitoid (*Trissolcus japonicus* (Ashmead)) as a classical biological control agent against *H. halys* in countries where it has been detected (Rice et al., 2014). In fact, two adventive introductions into the USA already occurred (Talamas, Johnson, & Buffington, 2015; Talamas, Herlihy, et al., 2015; Milnes et al., 2016).

Interspecific interactions between parasitoids can influence community structure which is important, not only from an ecological but also from a practical (biological control) perspective (Boivin & Brodeur, 2006). A thorough understanding of these interactions is critical to estimate the potential direct and indirect effects, positive or negative, associated with the introduction of the exotic parasitoid against an exotic pest. Although in‐host competition between parasitoid larvae can have negative consequences [e.g., death, reduced fitness (Godfray, 1994; Boivin & Brodeur, 2006)], there may be benefits in the form of interspecific facilitation (Cusumano, Peri, & Colazza, 2016; Poelman et al., 2014). For example, secretions injected by heterospecific or conspecific parasitoid females ovipositing into the same host may act synergistically to overcome the host immune response, or teratocytes released by one species may provide additional nutritional resources for the other species, thereby facilitating the development of the competitively superior parasitoid.

There is little information regarding interspecific interactions between native and exotic egg parasitoids associated with Pentatomidae, so in this study we determined the outcomes of larval competitive interactions between the exotic *T. japonicus* and the native *T. cultratus,* which might naturally occur following either an intentional introduction of *T. japonicus* as a biological control agent for *H. halys* in Europe, or an accidental one. This host–parasitoid model system presents an excellent opportunity to assess the possibility of different interspecific interactions (e.g., competition, facilitation), between a native and exotic parasitoid on an invasive host (*H. halys*), and evaluate the impacts of these interactions from basic and applied perspectives.

## 2 Materials and Methods

### 2.1 Stinkbug rearing

A colony of *H. halys* was established from individuals collected in Zurich, Switzerland, in 2012 and maintained in continuous rearing on a diet of organic beans (*Phaseolus vulgaris* L.) and corn (*Zea mays* L) at 26°C, 70%RH, 16L:8D photoperiod in Bug Dorms (50 × 50 × 50, Mega View). Fresh‐cut branches of cherry (*Prunus avium* L.), buckthorn (*Rhamnus* sp.) and hazelnut (*Corylus* sp.) were also provided when available. Strips of black mesh were provided as oviposition substrate. Eggs were collected daily and maintained under the same conditions.

### 2.2 Parasitoid rearing

*Trissolcus japonicus*, originally collected around Beijing (China), were maintained on fresh *H. halys* egg masses. *Trissolcus cultratus*, collected from frozen, sentinel *H. halys* egg masses in the Delémont valley (Canton Jura, Switzerland), were maintained on frozen (at −80°C and then thawed 30 min prior to use) *H. halys* egg masses. Parasitoids (mated, ≥2 days old) were held in a clear plastic container (10 cm diameter, 5 cm height) with 10% honey water solution as a food source and 8–10 fresh or frozen *H. halys* egg masses provided twice a week. Parasitized egg masses were kept at 26°C, 60% humidity, and 16L:8D photoperiod.

Upon the initial establishment of the laboratory colonies of *T. japonicus* and *T. cultratus*, specimens were sent to E. Talamas (Smithsonian Institution, Washington, DC, USA) and M. C. Bon (USDA‐ARS European Biological Control Laboratory, Montferrier sur Lez, France) for morphological and molecular identification, respectively.

### 2.3 Time‐course developmental study

All assays were carried out in plastic Petri plates (5 × 1 cm) into which females were introduced for the duration of the experiment, and observations were made (using a stereomicroscope) between 0800 and 1300 h at 26°C, 60% relative humidity, and 16L:8D photoperiod. Randomly selected *T. japonicus* females (4 days old, mated, naïve) were offered fresh (<24 h) *H. halys* egg masses (12 eggs/mass) and observed until all the eggs in the mass were parasitized (as indicated by marking behavior) or until female left the egg mass for >10 min. *Trissolcus cultratus* females (4 days old, mated, naïve) were provided fully parasitized egg masses (*n* = 20 per time interval) that were attacked by *T. japonicus* 0 hr, 1, 2, 3, 4, or 5 days earlier and observed until they had parasitized the entire egg mass, or left the mass for >10 min. As controls, fresh and frozen *H. halys* egg masses (12 eggs/mass) of the corresponding ages were offered to *T. cultratus* females to determine the effect of host age and host necrosis on acceptance and suitability for development (*n* = 10 per time interval). For frozen egg controls, the eggs were reared for 0, 1, 2, 3, 4, and 5 days prior to being frozen and exposed to the parasitoid. This approach ensured that parasitoid was provided hosts at different stages of embryonic development. The number of eggs attempted (drilled) and parasitized (marked) by each *T. cultratus* female was recorded. Egg masses were kept for 3 weeks and all adult parasitoids obtained were identified to species based on morphological characteristics including facial striae and clypeal setae (Talamas, Johnson, & Buffington, 2015; Talamas, Herlihy, et al., 2015a, b).

### 2.4 Egg mass (patch) guarding behavior

To determine how long *T. japonicus* females guard parasitized egg masses, 20 egg masses (28 ± 1 eggs/egg mass) were exposed to randomly selected 4 days old, mated, naïve females in small (5 × 1 cm) plastic Petri plates inside large Petri dish arenas (15 × 2 cm). Once the egg mass was parasitized, a second small Petri plate with a fresh egg mass was placed in the arena and the female's guarding behavior was verified every 6 hr until she left the parasitized egg mass.

### 2.5 Statistical analysis

The proportions of eggs drilled and marked were arcsine‐transformed (to normalize the data) and analyzed with a two‐way ANOVA followed by a Tukey post hoc test. In cases where the effects of egg mass age and/or state were significant, further one‐way ANOVA analyses with Tukey post hoc tests were carried out. The developmental outcomes from parasitized eggs for each egg state were analyzed using separate χ^2^ tests, with the *p*‐value for each cell calculated based on resulting residuals, and compared to Bonferroni corrected *p*‐values (indicating if the proportion of each developmental outcome was significantly different from a mean proportion of that outcome across the egg age or egg state). All statistical analyses were carried out using SPSS (v.23) statistical software.

## 3 Results

### 3.1 Host acceptance behavior

**Figure 2:**
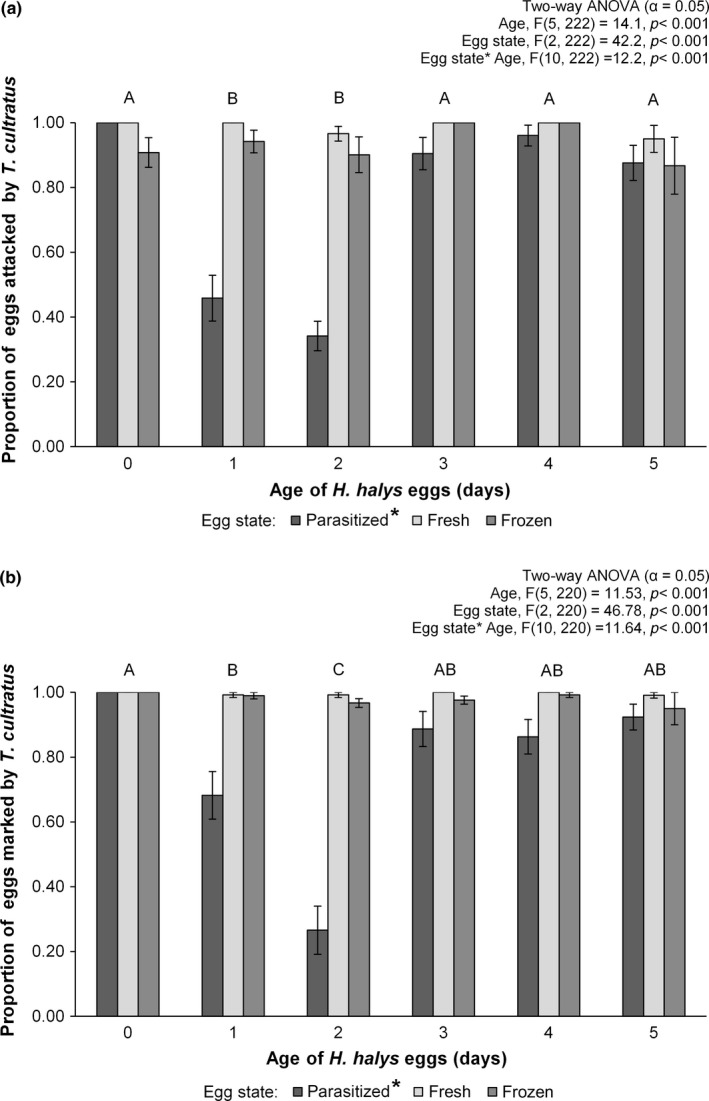
Mean proportion (±SE) of (unparasitized) fresh or frozen *Halyomopha halys* egg masses, as well as fresh egg masses previously parasitized by *Trissolcus japonicus* which were (a) drilled and (b) marked by *Trissolcus cultratus* as a function of age/time since parasitization by *T. japonicus*. Bars not sharing the same letters are statistically different based on Tukey's post hoc test

*Trissolcus cultratus* females readily attacked and marked *H. halys* egg masses, but the relative proportions of drilled and marked eggs varied based on age and state (i.e., parasitized, fresh, frozen) of the egg masses (Figure 2a,b).

The proportion of control *H. halys* egg masses that *T. cultratus* females drilled (fresh: *F*_(5,59)_ = 1.56, *p* = .19; frozen: *F*_(5,59)_ = 1.51, *p* = .20) or marked (fresh: *F*_(5,59)_ = 0.60, *p* = .70; frozen: *F*_(5,59)_ = 0.86, *p* = .51) did not differ as a function of egg mass age, averaging >93% in all cases. A similar trend was observed when females were provided 0‐, 3‐, 4‐, and 5‐day‐old parasitized*. H. halys* eggs (Figure 2a,b). However, significantly fewer egg masses parasitized by *T. japonicus* one and two days prior to testing were drilled (*F*_(5,119)_ = 37.1, *p* < .001) and marked (*F*_(5,117)_ = 26.4, *p* < .001; Figure 2a,b) by *T. cultratus* females.

### 3.2 Developmental success and interspecific larval competition

**Figure 3:**
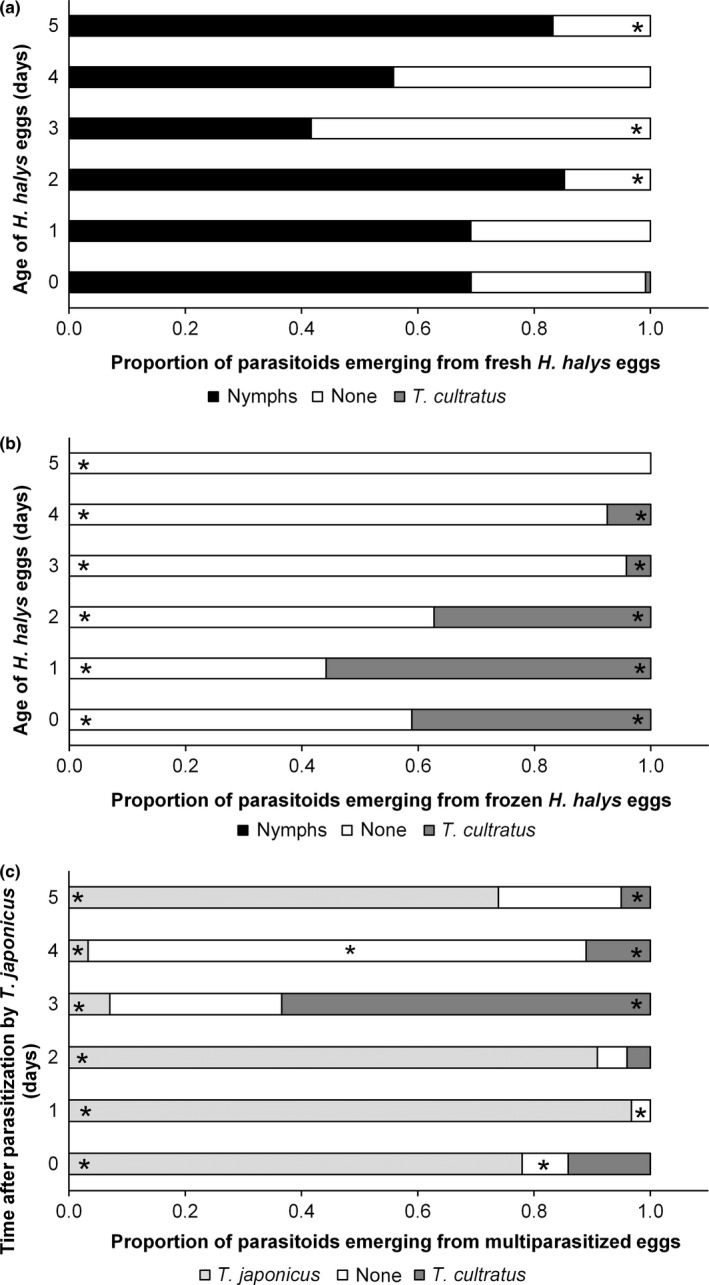
Mean proportion of *Trissolcus japonicus, Trissolcus cultratus, Halyomopha halys* nymph or nothing emerging from different aged *H. halys* eggs that were (a) fresh, (b) frozen or (c) multiparasitized by *T. japonicus* and *T. cultratus* at different time intervals (ages). Asterisks (*) indicate proportions of each outcome for fresh, frozen, and multiparasitized eggs that is significantly different from a mean proportion of that outcome across the egg age (χ^2^ tests with Bonferroni corrections)

The proportion of *T. cultratus* larvae successfully developing in fresh (χ^2^_(10, *N *= 707)_ = 76.2, *p* < .001) and frozen (χ^2^_(5, *N *= 663)_ = 169.1, *p* < .001) *H. halys* eggs varied as a function of egg mass age. Less than 1% of <24‐h‐old eggs supported the development of *T. cultratus*, while no parasitoids emerged from those >24 h (Figure 3a), despite observed drilling and marking behavior (Figure 2a,b). However, parasitoid attack did reduce the number of *H. halys* that emerged. In contrast, some *T. cultratus* (4%–54%) successfully developed in 0‐ to 4‐day‐old frozen egg masses although the proportion declined with age of the egg mass (Figure 3b).

In contrast to the pattern seen with fresh, unparasitized *H. halys* eggs (Figure 3a), some *T. cultratus* were able to successfully complete development when exploiting eggs that had previously been parasitized by the exotic *T. japonicus* (Figure 3c). The relative developmental success of both parasitoids was influenced by the time elapsed between *T. japonicus* and *T. cultratus* females ovipositing (χ^2^_(10, *N *= 942)_ = 746.8, *p* < .001). If the time delay was 2 days or less, then at least 75% of the hosts gave rise to *T. japonicus* while <15% gave rise to *T. cultratus*. A similar pattern was observed when the time delay between oviposition was 5 days, but if there was a 3‐day difference, then the majority of parasitized host eggs produced *T. cultratus*. Interestingly, when there was a 4‐day time difference between oviposition, very few parasitoids of either species emerged, whereas in the 5‐day interval treatment, a high proportion of *T. japonicus* adults emerged (Figure 3c).

### 3.3 Egg mass guarding behavior

Following parasitization of fresh *H. halys* egg masses (28 eggs/mass), 90% of *T. japonicus* females guarded the masses for <6 or 12 hr and were found either parasitizing a second egg mass, or exploring Petri plates in search of alternate recourses. Only a single female remained on the parasitized egg mass for 24 hr.

## 4 Discussion

The intertrophic level effects of introducing a novel potential host into an ecosystem will depend in part on the “decision‐making” behaviors of parasitoids that are based on the use of reliable cues during host habitat location, host location, host acceptance, and host suitability (Vinson, 1976) to maximize reproductive success (Schlaepfer et al., 2005; Williams & Nichols, 1984). Thus, if a new host is unsuitable for development, this could result in an evolutionary trap for parasitoids unless at some stage in the foraging process females avoid unsuitable hosts (Phillips & Shine, 2004) or can overcome the defensive barriers of the host (Keeler & Chew, 2008). Our results support the hypothesis that the introduction of *H. halys* represents a potential evolutionary trap that could impact the diversity and population dynamics of native Pentatomidae and their scelionid parasitoids (Abram et al., 2014), given that native parasitoids accept *H. halys* as a host but fail to complete development in healthy eggs (Abram et al., 2014; Haye et al., 2015). It has also been observed that introduced species often facilitate one another's establishment, resulting in an “invasional meltdown” (Simberloff, 2006; Simberloff & Von Holle, 1999), and even the introduction of a beneficial species for biological control may interact with other introduced species in a way that negatively impacts native species (Howarth, 1985; Messing, Roitberg, & Brodeur, 2006; Schellhorn et al., 2002). The facilitation of native species by nonindigenous ones has been largely overlooked (Rodriguez, 2006) as has interspecific facilitation among parasitoids (Cusumano et al., 2016).

**Figure 4:**
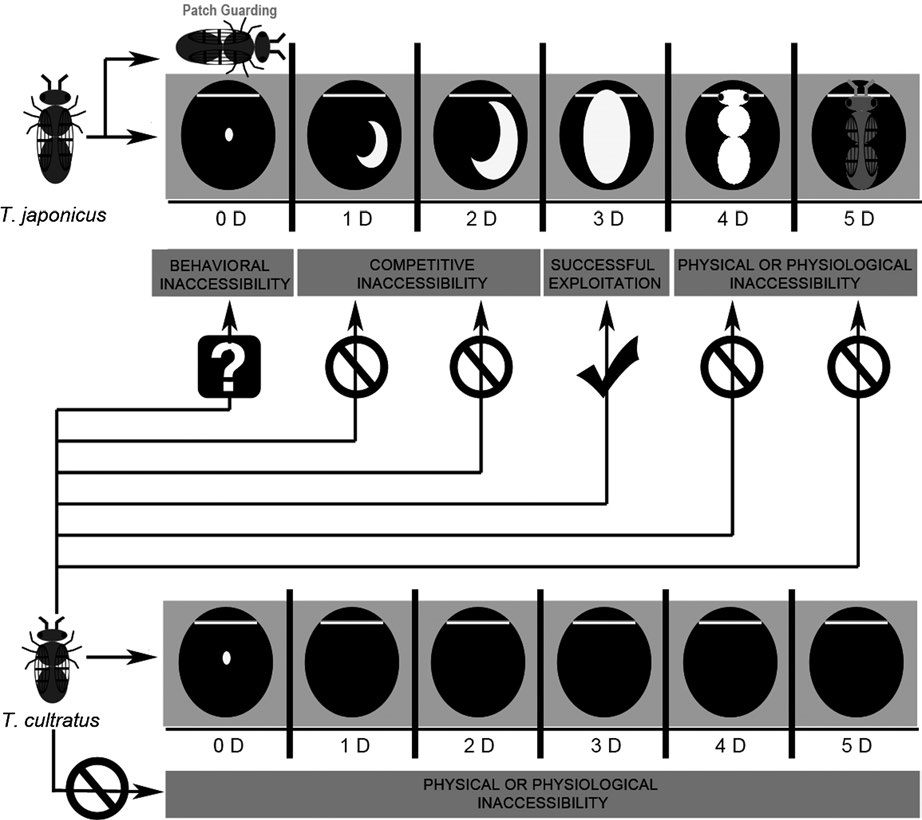
General schematic showing the temporal pattern of suitability of parasitized and unparasitized *Halyomopha halys* eggs for *Trissolcus cultratus* as a function of their age

Our results indicate that the introduction of *T. japonicus* as a biological control agent against *H. halys* may actually provide native *Trissolcus* species a partial escape from the evolutionary trap created by the presence of *H. halys*, because, under certain conditions, *T. cultratus* may successfully develop in *H. halys* eggs previously attacked by *T. japonicus* (Figure 3c). To our knowledge, this is the first example of interspecific facilitation among egg parasitoids and the first example of a secondary invader (*T. japonicus*) potentially facilitating the use of a primary invader (*H. halys*) as host by a native species (*T. cultratus*). The time window for this “invasional lifeline” will be quite narrow for several reasons (Figure 4) and will only exist where *T. japonicus* becomes well established in areas where *H. halys* are found.

We assume that factors injected by *T. japonicus* females to overcome host defences allow *T. cultratus* to exploit *H. halys* as a primary parasitoid in a small percentage of attacks that occur immediately after *T. japonicus* has oviposited (see day 0 in Figure 3c). However, as *T. japonicus* females remained on the egg mass for 6–12 hr following oviposition (patch guarding), the likelihood of *T. cultratus* females finding recently parasitized, unguarded *H. halys* egg masses would be low under natural conditions.

The fact that *T. cultratus* females attacked and marked significantly fewer *H. halys* eggs 1 and 2 days after they had been previously attacked by *T. japonicus* when compared to day 0 (Figure 2c), suggested that they are responding to the presence of the developing *T. japonicus* larvae rather than the presence of a marking pheromone. Avoiding eggs at this stage of *T. japonicus* development would be adaptive, as few or no *T. cultratus* adults emerged from those that were attacked (Figure 3c), suggesting that the early *T. japonicus* larval instars equipped with sickle‐shaped mandibles are superior competitors and effectively eliminate eggs or newly hatched *T. cultratus* larvae. In contrast, *T. cultratus* was quite successful when attacking *H. halys* eggs containing fully grown *T. japonicus* larvae (e.g., 3 days following the initial parasitization by *T. japonicus*; Figure 3c). In this case, *T. cultratus* acts as a facultative hyperparasitoid on *T. japonicus*, a tactic used by some egg parasitoids in an environment with limited, unexploited resource (Brodeur, 2000). For example, the generalist egg parasitoid, *Ooencyrtus telenomicida* (Vassiliev) (Hymenoptera: Encyrtidae), is capable of exploiting pentatomid egg masses either as a primary parasitoid or as a facultative hyperparasitoid when eggs have already been parasitized by *Trissolcus basalis* (Wollaston) (Cusumano et al., 2013).

Even though *T. cultratus* females readily accept and mark eggs attacked 4 and 5 days previously by *T. japonicus* (Figure 2a,b), they are no longer suitable hosts (Figure 3c). This may be due to the salivary secretions produced by parasitoid larvae just prior to pupation that solidify to provide protection from foreign bodies (Safavi, 1968) and the subsequent sclerotization of the pupal exoskeleton (Volkoff & Colazza, 1992). Interestingly, this is similar to the situation observed when *T. cultratus* females were provided *H. halys* eggs that had been frozen 3–5 days after being laid (Figure 3b); while eggs were attacked and marked (Figure 1), very few progeny are produced (Figure 3) suggesting that the advanced nymphal stages of *H. halys* were unsuitable for *T. cultratus* larvae. This effect of host developmental stage has been observed in other *Trissolcus* species, with females attacking all developmental stages of host eggs and successful parasitism decreasing significantly in eggs >3 days old (Awadalla, 1996; Kivan & Kilic, 2005).

As noted, facultative hyperparasitism has not been extensively studied and it is possible that many scelionid parasitoids associated with stinkbugs use this strategy. Thus, *T. japonicus* may also develop as a facultative hyperparasitoid on congeneric species and could exploit native scelionids developing within other pentatomid species. Intraguild predation in the form of facultative hyperparasitism may reduce the success of a biological control program (Boivin & Brodeur, 2006; Messing et al., 2006) or promote conservation and stabilization of natural communities (Müller & Brodeur, 2002). Consequently, we suggest that the possibility of facultative hyperparasitism should be investigated when assessing risk factors to native species following the introduction of exotics for classical biological control.

The actual impact of this potential evolutionary trap will depend on a number of other factors, such as (1) the relative densities of the introduced and indigenous host egg masses and (2) the degree to which native parasitoids prefer native hosts over introduced ones, as the impact would be lesser if females of different species discriminate in their selection of oviposition sites. Furthermore, as *T. japonicus* is a generalist parasitoid of Pentatomidae, it could exploit native stinkbug egg masses following its introduction as a classical biological control agent against *H. halys* (Talamas, Johnson, & Buffington, 2015; Talamas, Herlihy, et al., 2015). If this is the case, one would predict that interspecific competition could result in a significant decline in native parasitoid populations, especially if, under field conditions, indigenous species readily accept *H. halys* eggs. However, as noted above for native species, the outcome will also depend on whether *T. japonicus* preferentially oviposit in *H. halys* egg masses. Clearly, the preference of foraging native parasitoids and *T. japonicus* must be evaluated in order to assess the real impact that *H. halys* will have on food webs following its introduction into new ecosystems. In the long term, it is possible that native parasitoids successfully developing on *H. halys* eggs previously parasitized by *T. japonicus* will learn to recognize and seek out cues indicative of both the acceptability and developmental suitability of *H. halys* as a host. It is therefore essential that all potential interactions between native and exotic egg parasitoids be investigated as this information will be vital to predict potential ecological outcomes, including the efficacy of biological control programs against *H. halys*.

## Conflict of Interest

None declared.
